# Supplementary material for: LLM-FMS: A fine-grained dataset for functional movement screen action quality assessment
Source: PLoS One. 2025 Mar 11;20(3):e0313707. doi: 10.1371/journal.pone.0313707 (PMC11896072; doi:10.1371/journal.pone.0313707)
Supplement: S1 Text — (PDF) [file pone.0313707.s002.pdf]

m01-Deep Squat (Floor)

- The angle of knee joint is the smallest.

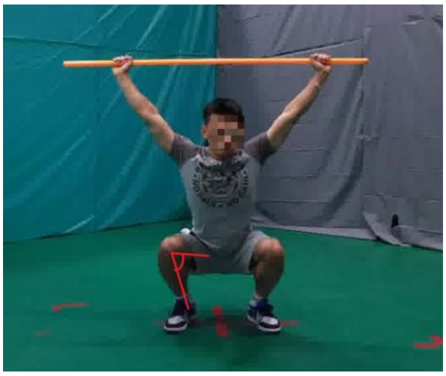

m02- Deep Squat (Plank)

same as m01.

m03- Hurdle Step (Left)

- The angle of hip joint on the non-supporting side is the smallest (the angle between the blue line segment in the figure below).

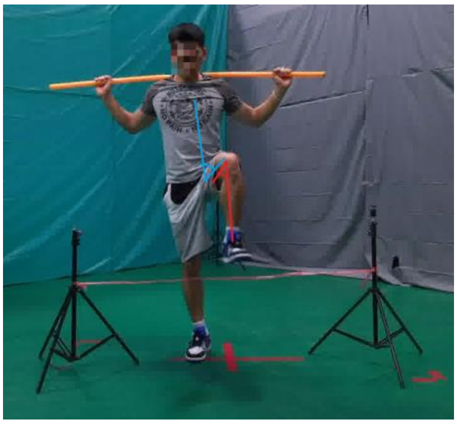

m04- Hurdle Step (Right)

same as m03.

m05- Inline Lunge (Left)

- The distance between anterior ankle joint and posterior knee joint is the shortest.

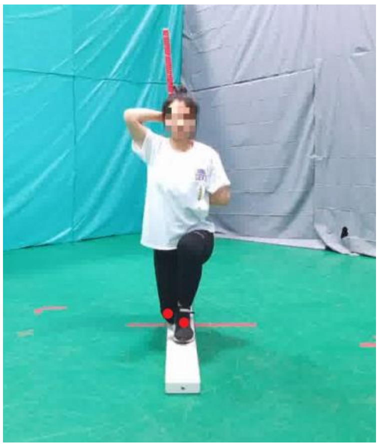

m06- Inline Lunge (Right)

same as m05.

m07- Shoulder Mobility (Left)

- The distance between the left and right wrist is the shortest.

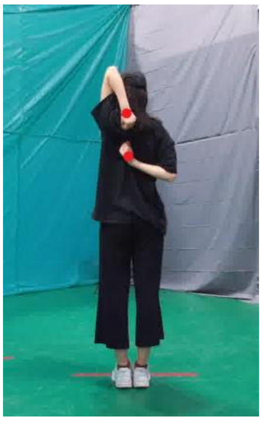

m08- Shoulder Mobility (Right)

same as m07.

m09- Active Straight-Leg Raise (Left)

- The angle between the legs is the biggest.

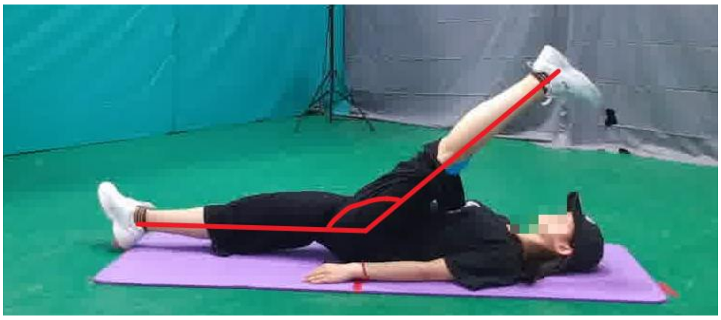

m10- Active Straight-Leg Raise (Right)

same as m09.

m11- Trunk Stability Push-Up

- The shoulder and wrist joints have the greatest distance.

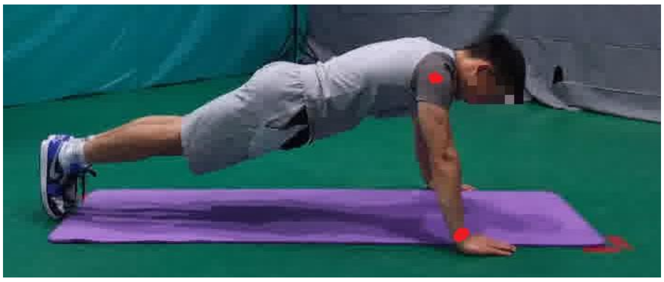

m12- Rotary Stability (Ipsilateral&Left)

- The distance between the raised side wrist and the knee joint is the shortest.

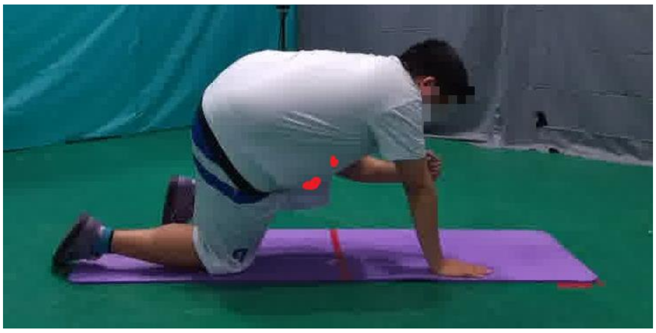

m13- Rotary Stability (Ipsilateral&Right)

same as m12.

m14- Rotary Stability (Contralateral&Left upper limb)

- The raised wrist and knee joints have the shortest distance.

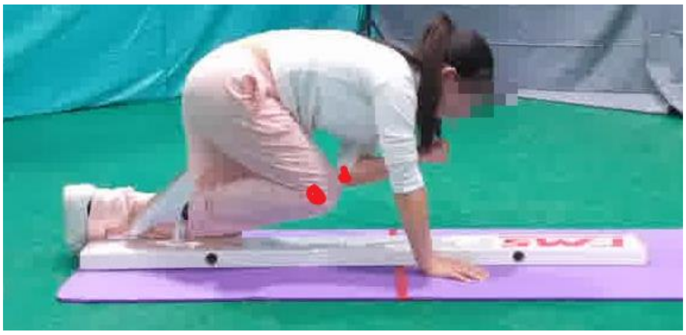

m15- Rotary Stability (Contralateral&Right upper limb)

same as m14.
