# Supplementary material for: LLM-FMS: A fine-grained dataset for functional movement screen action quality assessment
Source: PLoS One. 2025 Mar 11;20(3):e0313707. doi: 10.1371/journal.pone.0313707 (PMC11896072; doi:10.1371/journal.pone.0313707)
Supplement: S2 Text — (PDF) [file pone.0313707.s003.pdf]

Human skeleton key points (COCO’s 17 key joints)

Index	Joint Name
0	Nose
1	Left eye
2	Right eye
3	Left ear
4	Right eat
5	Left shoulder
6	Right shoulder
7	Left elbow
8	Right elbow
9	Left wrist
10	Right wrist
11	Left hip
12	Right hip
13	Left knee
14	Right knee
15	Left ankle
16	Right ankle

FMS

M01/M02-Deep Squat-Side view

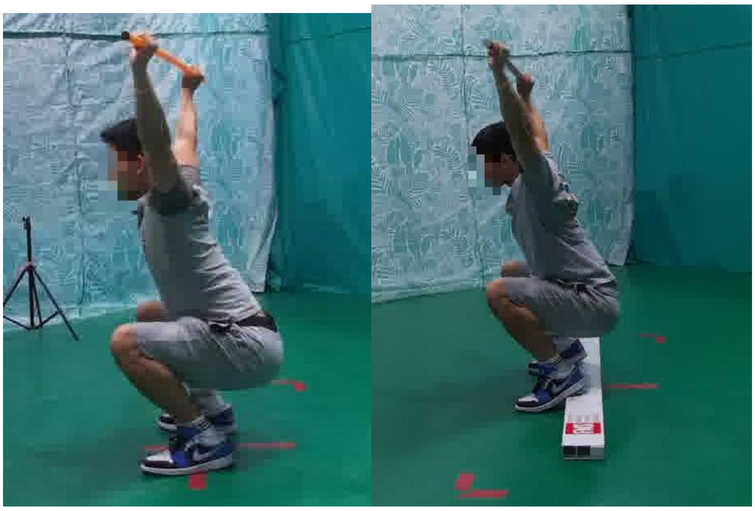

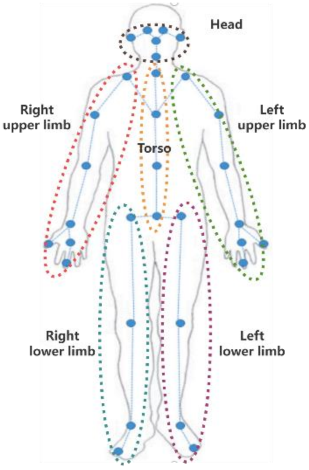

Angel 1: Standard angle range is [0, 10];

Index	Definition	Classification	Body part
Angle 1: Trunk-calf angle	The angle between the trunk and the calf.	Parallel (Standard)	Trunk, Left/Right lower limb
		Not parallel (Non-Standard)	
Position 1: Hip height	The height position relationship of the hip relative to the knee.	Higher than (Non-Standard)	Left/Right lower limb
		Equal to (Standard)	
		Lower than (Standard)	
Position 2: Wrist position	The horizontal position relationship of the wrist relative to the knee.	The left (Non-Standard)	Left/Right upper limb, Left/Right lower limb
		Equal to (Standard)	
		The right (Standard)	

M03/M04-Hurdle Step-Front view

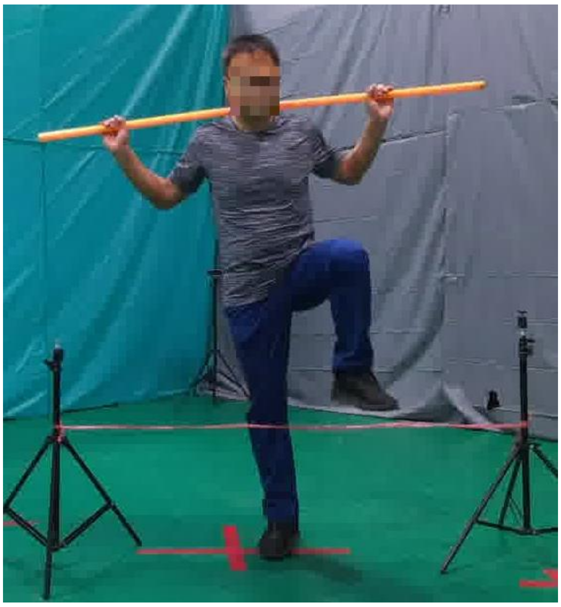

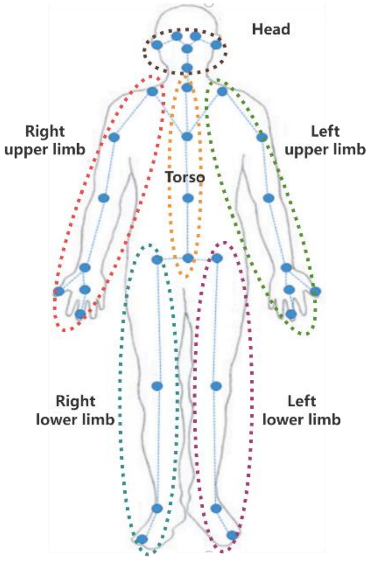

Angle 1: Standard angle range is [0, 10];

Angle 2: Standard angle range is [80, 90];

Angle 3: Standard angle range is [80, 90];

Positional 1: The standard positional relationship requires the ankle on the non-supported side to be higher than the knee on the supported side.

Index	Definition	Classification	Body part
Angle 1: Wrist connection angle	The angle between the line of the wrist joint and the horizontal plane.	Parallel (Standard)	Left/Right upper limb
		Not parallel (Non-Standard)	
Angle 2: Tilt angle of the support leg	The angle between the support leg and the horizontal plane.	Vertical (Standard)	Right lower limb
		Not vertical (Non-Standard)	
Angle 3: Tilt angle of the non-supporting shank	The angle between the lower leg on the non-supported side and the horizontal plane.	Vertical (Standard)	Left lower limb
		Not vertical (Non-Standard)	

Position 1: Height of the non-supporting foot	The height position of the ankle on the non-support side relative to the knee joint of the support leg.	Higher than (Non-Standard)	Left/Right lower limb
		Equal to (Standard)	
		Lower than (Standard)	

M05/M06-Inline Lunge-Side view

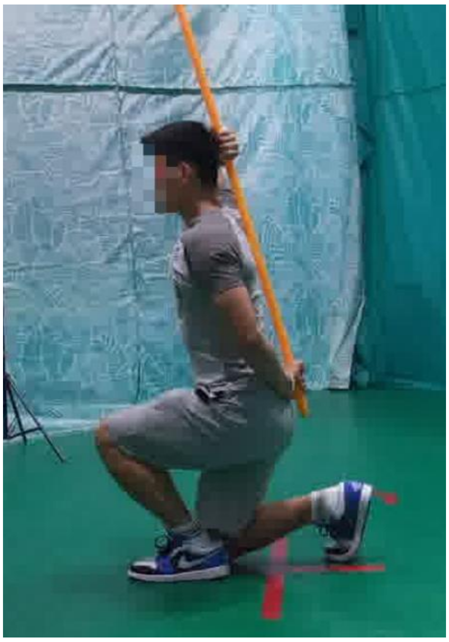

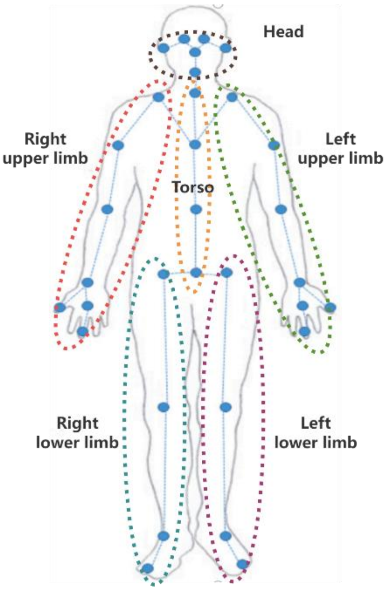

Angle 1: The angle between the torso and the vertical axis，standard angle range is [0, 10];

Distance 1: The distance between the knee joint of the back leg and the ankle joint of the front leg, the standard distance range is [0, 5].

Position 2:

Index	Definition	Classification	Body part
Angle 1: The inclination angle of the torso	The angle between the trunk and the vertical axis.	Parallel (Standard)	Torso
		Not parallel (Non-Standard)	

Distance 1: Knee-ankle spacing	Distance between the knee joint of the posterior leg and the ankle joint of the anterior leg.	Close to (Standard)<br>Far (Non-Standard)	Left/Right lower limb
Position 2: Height of the back knee	Height of the knee joint of the posterior leg relative to the ankle joint of the anterior leg.	Close to (Standard)<br>Far (Non-Standard)	Left/Right lower limb

M07/M08-Shoulder Mobility-Front view

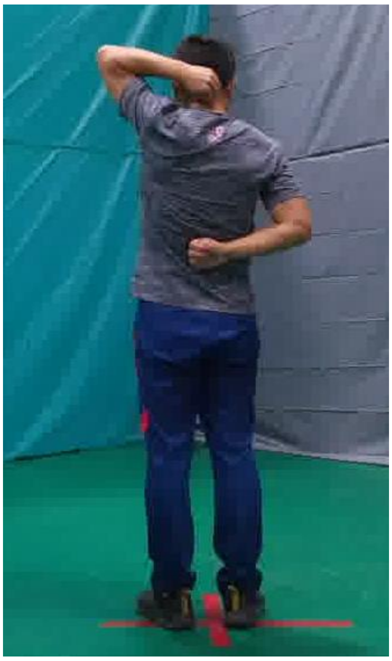

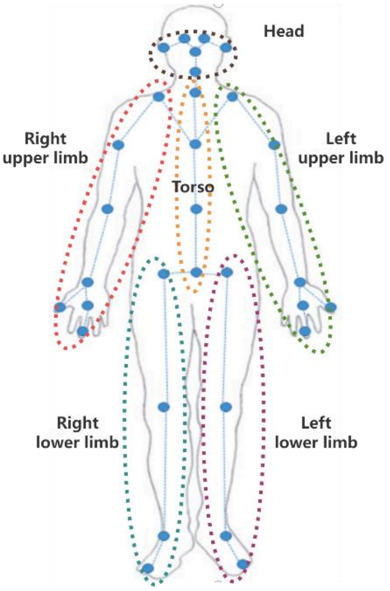

Distance 1: The distance between the left and right wrists. The standard distance should not exceed the length of one palm.

Index	Definition	Classification	Body part

Distance 1: Wrist spacing	The distance between the left and right wrists.	3 points: [0, 1]	Left/Right upper limb
		2 points: (1, 1.5)	
		1 point: (1.5, +∞)	

M09/M10-Active Straight-Leg Raise-Front view

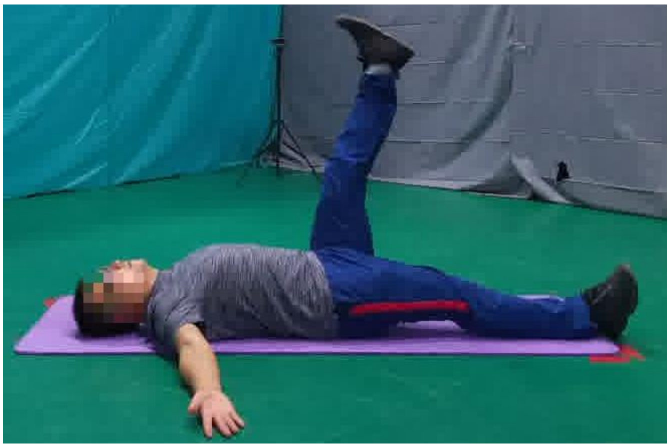

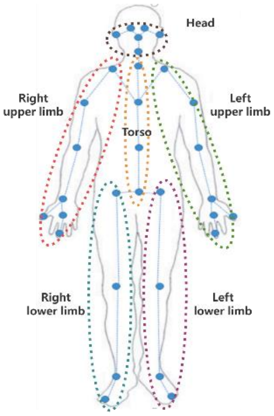

Position 1: The ankle joint on the non-support side makes a vertical line and lands on the ground in the position of the leg.

Index	Definition	Classification	Body part
Position 1: Ankle joint landing position	The ankle on the non-support side makes a vertical line and lands on the ground in the position of the leg.	3 points: [midpoint of thigh, head)	Left/Right lower limb
		2 points: [knee, midpoint of thigh)	
		1 point: (knee, ankle)	

M11- Trunk Stability Push-Up-Front view

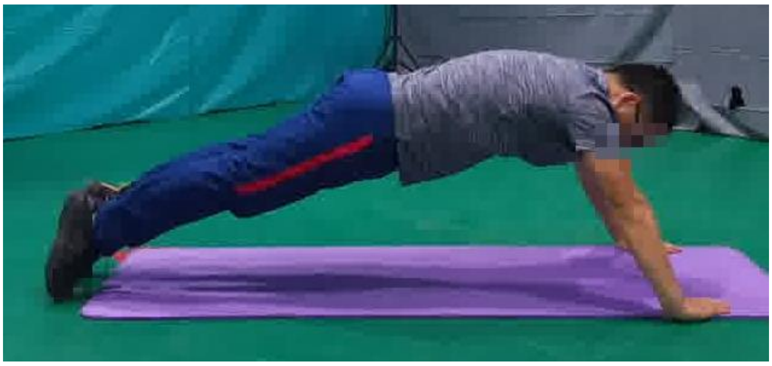

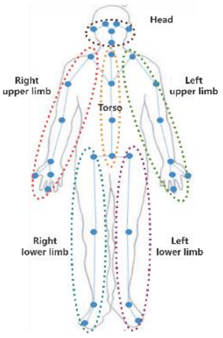

Position 1: Relationship between the position of the wrist and the vertex, upper neck, lower neck, and clavicle points on the horizontal axis.

Angle 1: Trunk-lower limb Angle， standard angle range is [0, 10];

Angle 2: Lower limb inclination, standard angle should be greater than 10 degress.

The scoring rules of this movement: The scoring of this movement is divided into 3 points, 2 points, 1 points and 0 points, which are as follows:

Score 3: For male users, the position 1 of the action is that the wrist is close to the overhead point, and the distance is within 0 ~ 3 cm. Angle 1 is the standard Angle, and Angle 2 is greater than 10 degrees; For female users, the position 1 of the action is that the wrist is close to the upper point of the neck, the distance is within 0 ~ 3 cm, Angle 1 is the standard Angle, Angle 2 is greater than 10 degrees, to get a full score of 3 points;

Score 2: For male users, the position 1 of action is that the wrist is close to the upper point of the neck, the distance is within 0 ~ 3 cm, Angle 1 is the standard Angle, Angle 2 is greater than 10 degrees; For female users, the position 1 of action is that the wrist joint is close to the clavicle point, the distance is within 0 ~ 3 cm, Angle 1 is the standard Angle, and Angle 2 is greater than 10 degrees.

Score 1: For male users, the position 1 of the action is that the wrist is close to the upper point of the neck, the distance is within 0 ~ 3 cm, and the Angle 2 is between 0 ~ 2 degrees; For female users, the position 1 of motion 1 is that the wrist joint is close to the clavicle point, the distance is within 0 ~ 3 cm, and the Angle 2 is between 0 ~ 2 degrees.

Score 0: The user feels pain.

Index	Definition	Classification	Body part
Position 1: Wrist position	Relationship between the position of the wrist and the nose, and the shoulder on the horizontal axis.	The tip of the nose and above.	Left/Right upper limb, torso
		[shoulder, nose]	
		Below the shoulder	
Angle 1: Trunk-lower limb Angle	Angle between trunk and lower limbs.	Parallel (Standard)	Torso, Left/Right lower limb
		Not parallel (Non-Standard)	
Angle 2: Lower limb inclination	Angle between thigh and horizontal plane.	Standard: >10 degrees	Torso, Left/Right lower limb
		Non-Standard: [0, 10)	

M12/13- Rotary Stability-Front view

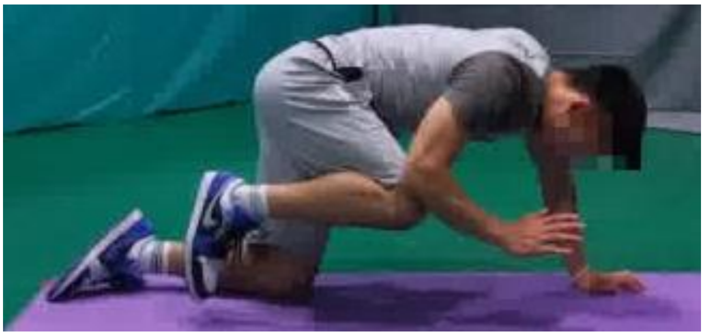

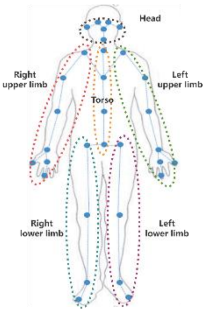

Distance 1: The distance between the elbow and knee on the ipsilateral side of the body., The standard distance range is [0, 5].

Index	Definition	Classification	Body part
Distance 1: Elbow-knee	The distance between the	Score 3: standard large	Left/Right upper limb, Left/Right

spacing	elbow and knee on the ipsilateral side of the body.	Score 2: non-standard range	lower limb

M14/15- Rotary Stability-Front view

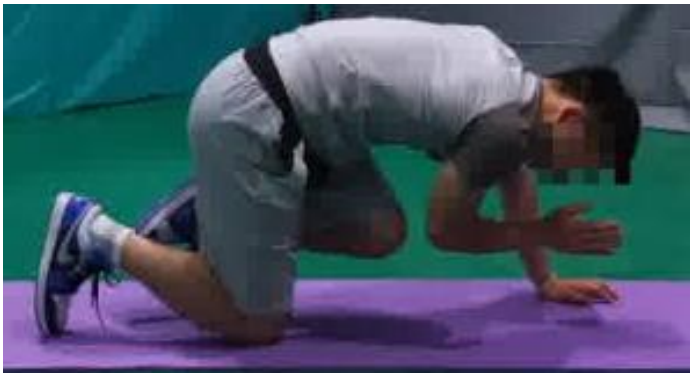

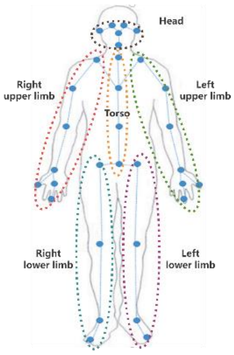

Distance 1: The distance between the elbow and knee on the contralateral side of the body., The standard distance range is [0, 5].

Index	Definition	Classification	Body part
Distance 1: Elbow-knee spacing	The distance between the elbow and knee on the contralateral side of the body.	Score 2: standard large	Upper limb, lower limb
		Score 1: non-standard range	
