# Supplementary material for: LLM-FMS: A fine-grained dataset for functional movement screen action quality assessment
Source: PLoS One. 2025 Mar 11;20(3):e0313707. doi: 10.1371/journal.pone.0313707 (PMC11896072; doi:10.1371/journal.pone.0313707)
Supplement: S3 Text — (PDF) [file pone.0313707.s004.pdf]

Human skeleton key points (COCO's 17 key joints)

Index	Joint Name
0	Nose
1	Left eye
2	Right eye
3	Left ear
4	Right eat
5	Left shoulder
6	Right shoulder
7	Left elbow
8	Right elbow
9	Left wrist
10	Right wrist
11	Left hip
12	Right hip
13	Left knee
14	Right knee
15	Left ankle
16	Right ankle

FMS

M01-Deep Squat-Side view (Floor)

Background knowledge:

We divided the human body into six parts: head, torso, left upper limb, right upper limb, left lower limb, and right lower limb. In order to better focus on the fine-grained performance of user actions. Deep Squat is the first action of Functional Movement Screen (FMS). In order to determine whether the user's action is standard, we used the following body angle and position information:

Angle 1: trunk-calf angle. Specifically the angle between the trunk (the line between the midpoint of the left and right hip joints and the midpoint of the left and right shoulder joints) and the leg tibia (the line between the left knee joint and the left ankle joint);

Position 1: hip height, specifically the height position relationship between the left hip joint and the left knee joint;

Position 2: Wrist position, specifically the horizontal position relationship between the left wrist joint and the left knee joint.

Scoring rules:

Trunk-calf angle: There are two situations of this angle: ① Standard angle: the trunk is parallel to the calf, and the angle range is [0, 5]. ② Non-standard angle: the trunk is not parallel to the calf, the angle range is (5, +∞).

Hip height: There are two types of hip height: ① Standard height: in the vertical axis direction, the hip joint is equal to or lower than the knee joint. ② Non-standard height: in the vertical axis direction, the hip is higher than the knee.

Wrist position: There are two cases of wrist position: ① Standard position: in the horizontal axis direction, the wrist joint and the knee joint position coincide, or the wrist joint on the right side of the knee joint. ② Non-standard position: in the horizontal axis direction, the wrist joint is on the left side of the knee joint.

Rating:

The movement is scored on a three-point scale of 3, 2, or 0, as follows:

Score 3 points: all angle information, height information and position information must fully meet

the standard;

Score 2: there is at least one angle information, height information or position information that does not meet the standard;

Score 0: the user feels pain.

To sum up, if you are a FMS tester, you first deep learn the above background knowledge and scoring rules, and then carefully reason about the angle information and position information of the following user actions:

<u>Trunk and calf parallel;</u>

<u>The hip was higher than the knee.</u>

<u>The wrist joint is to the right of the knee joint;</u>

<u>The user had no pain.</u>

Finally, you are asked to rate user actions. The output format is shown in the following example:

1. **Movement score: 3 points.**
2. **Main body parts: left upper limb, right upper limb, trunk, left lower limb, and right lower limb.**
3. **Evaluation criteria: the trunk-calf angle meets the standard interval; The hip joint of the lower limb was higher than the knee joint, which met the standard. The position of the wrist joint was on the right side of the knee joint, which met the criteria. The user had no pain.**
4. **Give the reasoning process of user angle and location information one by one:**
   a) **trunk-calf angle: meet the standard; Inference process: The trunk-calf angle is 1 degree, which is in the standard angle interval, indicating that the user's torso and calf are kept parallel;**
   b) **hip height: meet the standard; The reasoning process: the hip point was lower than the knee point, which met the standard position requirements.**
   c) **Wrist position: meet the standard; Reasoning process: The wrist joint is on the right side of the knee joint and meets the standard position requirements.**
5. **The user's body may have functional and joint flexibility problems.**

M02-Board deep squat-Side view

Background knowledge:

We divided the human body into six parts: head, torso, left upper limb, right upper limb, left lower

limb, and right lower limb. In order to better focus on the fine-grained performance of user actions. Board deep Squat is the first action (reduced order version) of Functional Movement Screen (FMS). In order to determine whether the user's action is standard, we used the following body angle and position information:

Angle 1: trunk-calf angle, specifically the angle between the trunk (the line between the midpoint of the left and right hip joints and the midpoint of the left and right shoulder joints) and the leg tibia (the line between the left knee joint and the left ankle joint);

Position 1: hip height, specifically the height position relationship between the left hip joint and the left knee joint;

Position 2: Wrist position, specifically the horizontal position relationship between the left wrist joint and the left knee joint.

Scoring rules:

Trunk-calf angle: There are two situations of this angle: ① Standard angle: the trunk is parallel to the calf, and the angle range is [0, 5]. ② Non-standard angle: the trunk is not parallel to the calf, the angle range is (5, +∞).

Hip height: There are two types of hip height: ① Standard height: in the vertical axis direction, the hip joint is equal to or lower than the knee joint. ② Non-standard height: in the vertical axis direction, the hip is higher than the knee.

Wrist position: There are two cases of wrist position: ① Standard position: in the horizontal axis direction, the wrist joint and the knee joint position coincide, or the wrist joint on the right side of the knee joint. ② Non-standard position: in the horizontal axis direction, the wrist joint is on the left side of the knee joint.

Rating:

The movement is scored on a three-point scale of 2, 1, or 0, as follows:

Score 2 points: all angle information, height information and position information must fully meet the standard;

Score 1: there is at least one angle information, height information or position information that does not meet the standard;

Score 0: the user feels pain.

To sum up, if you are a FMS tester, you first deep learn the above background knowledge and scoring rules, and then carefully reason about the angle information and position information of the following user actions:

Trunk and calf parallel;

The hip was higher than the knee.

The wrist joint is to the right of the knee joint;

The user had no pain.

Finally, you are asked to rate user actions. The output format is shown in the following example:

1. **Movement score: 2 points.**
2. **Main body parts: left upper limb, right upper limb, trunk, left lower limb, and right lower limb.**
3. **Evaluation criteria: the trunk-calf angle meets the standard interval; The hip joint of the lower limb was higher than the knee joint, which met the standard. The position of the wrist joint was on the right side of the knee joint, which met the criteria. The user had no pain.**
4. **Give the reasoning process of user angle and location information one by one:**
    a) **trunk-calf angle: meet the standard; Inference process: The trunk-calf angle is 1 degree, which is in the standard angle interval, indicating that the user's torso and calf are kept parallel;**
    b) **hip height: meet the standard; The reasoning process: the hip point was lower than the knee point, which met the standard position requirements.**
    c) **Wrist position: meet the standard; Reasoning process: The wrist joint is on the right side of the knee joint and meets the standard position requirements.**
5. **The user's body may have functional and joint flexibility problems.**

M03-Left hurdle step-Front view

Background knowledge:

We divided the human body into six parts: head, torso, left upper limb, right upper limb, left lower limb, and right lower limb. In order to better focus on the fine-grained performance of user actions. The left leg Hurdle Step is the second action of the Functional Movement Screen (FMS), with the left leg moving forward over the hurdle. To determine whether a user's hurdle step is standard or not, we used the following body angle and position information:

Angle 1: wrist connecting line angle, specifically the angle between the left and right wrist joint connecting line and the horizontal axis;

Angle 2: the tilt angle of the support leg, specifically the angle between the support leg (the connection line of the hip joint and the ankle joint) and the horizontal plane;

Angle 3: the angle of raising the lower leg, specifically the angle between raising the lower leg (the connection line of the knee joint and the ankle joint) and the horizontal plane;

Position 1: The height of the raised foot, specifically the position relationship between the raised ankle joint and the knee joint of the supporting leg in the vertical axis direction.

Among them, the support leg is the right leg, and the lift leg is the left leg.

Scoring rules:

Wrist connection Angle: There are two cases of this angle: ① Standard angle: the connection angle of the left and right wrist joints is parallel to the horizontal axis, the angle range is [0, 5]. ② Non-standard angle: the angle of the connecting line of the left and right wrist joints is not parallel to the horizontal axis, and the angle range is (5, +∞).

Incline angle of supporting leg: There are two kinds of angle: ① Standard angle: the connection line of hip joint and ankle joint of supporting leg is perpendicular to the horizontal axis. The angle range is [85, 90]. ② Non-standard angle: the connection lines of the hip joint and ankle joint of the supporting leg are not perpendicular to the horizontal axis, in which case the angle range is [0, 85].

The Angle of raising the lower leg: there are two kinds of angle: ① Standard angle: the line of raising the knee joint and ankle joint of the lower leg is perpendicular to the horizontal axis. The angle range is [85, 90]. ② Non-standard angle: the connection line of the knee joint and ankle joint of the raised leg is not perpendicular to the horizontal axis, and the range of this angle is [0, 85].

Foot lift height: There are two types of height: ① Standard height: in the vertical axis, the ankle joint is raised higher than the knee joint of the support leg. ② Non-standard height: in the vertical axis direction, the raised ankle joint is equal to or lower than the supporting leg knee joint.

Rating:

The movement is scored on a four-point scale of 3, 2, 1, and 0 as follows:

Score 3 points: all angle information and position information must be completely in line with the

standard;

Score 2: Position 1 must meet the standard, while any one or more of the first three body angle information and position information above does not meet the standard;

Score 1 point: as long as position 1 does not meet the criteria, score 1 point;

Score 0: the user feels pain.

To sum up, if you are a FMS tester, you first deep learn the above background knowledge and scoring rules, and then carefully reason about the angle information and position information of the following user actions:

The wrist connection angle was parallel to the horizontal axis.

The hip-ankle line of the support leg is not perpendicular to the horizontal axis.

The knee-ankle line of the raised leg is not perpendicular to the horizontal axis.

Lift the ankle joint above the knee joint of the support leg.

Finally, you are asked to rate user actions. The output format is as follows:

1. **Movement score: 2 points.**
2. **Main body parts: left upper limb, right upper limb, left lower limb and right lower limb.**
3. **Scoring basis: the wrist connection is not parallel to the horizontal axis and does not meet the standard interval; The supporting leg is perpendicular to the horizontal plane and meets the standard; Lift the leg perpendicular to the horizontal plane, in line with the standard; The raised foot was higher than the knee joint of the support leg, which met the standard; The user had no pain.**
4. **Give the reasoning process of user angle and location information one by one:**
    a) **Wrist connection angle: does not meet the standard; Inference process: The wrist connection angle is 6 degrees, which is not in the standard angle interval, indicating that the user's wrist connection is not parallel to the horizontal axis;**
    b) **Tilt angle of support leg: meet the standard; Reasoning process: the angle between the connection line of the hip joint and ankle joint of the support leg and the horizontal axis is 85 degrees, indicating that the user's support leg is perpendicular to the horizontal plane.**
    c) **Calf tilt angle: meet the standard; Reasoning process: The angle between the line of the knee joint and the ankle joint and the horizontal axis was 90 degrees, indicating that the leg was perpendicular to the horizontal plane.**

    d) **Height of lifting foot: meet the standard; Reasoning process: Raising the ankle joint above the knee joint of the support leg meets the standard position requirements.**

5. **The user's body may have functional and joint flexibility problems.**

M04-Right hurdle step-Front view

Background knowledge:

We divided the human body into six parts: head, torso, left upper limb, right upper limb, left lower limb, and right lower limb. In order to better focus on the fine-grained performance of user actions. The right-leg Hurdle Step is the second action of the Functional Movement Screen (FMS), with the right leg moving forward over the hurdle. To determine whether a user's hurdle step is standard or not, we used the following body angle and position information:

Angle 1: wrist connecting line angle, specifically the angle between the left and right wrist joint connecting line and the horizontal axis;

Angle 2: the tilt angle of the support leg, specifically the angle between the support leg (the connection line of the hip joint and the ankle joint) and the horizontal plane;

Angle 3: the angle of raising the lower leg, specifically the angle between raising the lower leg (knee joint and ankle joint are the connection line) and the horizontal plane;

Position 1: The height of the raised foot, specifically the position relationship between the raised ankle joint and the knee joint of the supporting leg in the vertical axis direction.

Among them, the support leg is the right leg, and the lift leg is the left leg.

Scoring rules:

Wrist connection Angle: There are two cases of this angle: ① Standard angle: the connection angle of the left and right wrist joints is parallel to the horizontal axis, the angle range is [0, 5]. ② Non-standard angle: the angle of the connecting line of the left and right wrist joints is not parallel to the horizontal axis, and the angle range is (5, +∞).

Incline Angle of supporting leg: There are two kinds of angle: ① Standard angle: the connection line of hip joint and ankle joint of supporting leg is perpendicular to the horizontal axis. The angle range is [85, 90]. ② Non-standard angle: the connection lines of the hip joint and ankle joint of the supporting leg are not perpendicular to the horizontal axis, the angle range is [0, 85].

The Angle of raising the lower leg: there are two kinds of angle: ① Standard angle: the line of

raising the knee joint and ankle joint of the lower leg is perpendicular to the horizontal axis. In this case, the angle range is [85, 90]. ② Non-standard angle: the connection line of the knee joint and ankle joint of the raised leg is not perpendicular to the horizontal axis, and the range of this angle is [0, 85].

Foot lift height: There are two types of height: ① Standard height: in the vertical axis, the ankle joint is raised higher than the knee joint of the support leg. ② Non-standard height: in the vertical axis direction, the raised ankle joint is equal to or lower than the supporting leg knee joint.

Rating:

The movement is scored on a four-point scale of 3, 2, 1, and 0 as follows:

Score 3 points: all angle information and position information must be completely in line with the standard;

Score 2: Position 1 must meet the standard, while any one or more of the first three body angle information and position information above does not meet the standard;

Score 1 point: as long as position 1 does not meet the criteria, score 1 point;

Score 0: the user feels pain.

To sum up, if you are a FMS tester, you first deep learn the above background knowledge and scoring rules, and then carefully reason about the angle information and position information of the following user actions:

<u>The wrist connection angle was parallel to the horizontal axis.</u>

<u>The hip-ankle line of the support leg is not perpendicular to the horizontal axis.</u>

<u>The knee-ankle line of the raised leg is not perpendicular to the horizontal axis.</u>

<u>Lift the ankle joint above the knee joint of the support leg.</u>

Finally, you are asked to rate user actions. The output format is as follows:

1. **Movement score: 2 points.**
2. **Main body parts: left upper limb, right upper limb, left lower limb and right lower limb.**
3. **Scoring basis: the wrist connection is not parallel to the horizontal axis and does not meet the standard interval; The supporting leg is perpendicular to the horizontal plane and meets the standard; Lift the leg perpendicular to the horizontal plane, in line with the standard; The raised foot was higher than the knee joint of the support leg, which met the**

standard; The user had no pain.

4. **Give the reasoning process of user angle and location information one by one:**
    a) **Wrist connection angle: does not meet the standard; Inference process: The wrist connection angle is 6 degrees, which is not in the standard angle interval, indicating that the user's wrist connection is not parallel to the horizontal axis;**
    b) **Tilt angle of support leg: meet the standard; Reasoning process: the angle between the connection line of the hip joint and ankle joint of the support leg and the horizontal axis is 85 degrees, indicating that the user's support leg is perpendicular to the horizontal plane.**
    c) **Calf tilt angle: meet the standard; Reasoning process: The angle between the line of the knee joint and the ankle joint and the horizontal axis was 90 degrees, indicating that the leg was perpendicular to the horizontal plane.**
    d) **Height of lifting foot: meet the standard; Reasoning process: Raising the ankle joint above the knee joint of the support leg meets the standard position requirements.**
5. **The user's body may have functional and joint flexibility problems.**

M05-Left Inline Lunge-Side view

Background knowledge:

We divided the human body into six parts: head, torso, left upper limb, right upper limb, left lower limb, and right lower limb. In order to better focus on the fine-grained performance of user actions. The Inline Lunge with the left leg in front is the third Movement of the Functional Movement Screen (FMS), with the left leg stepping forward. To determine whether the user's lunge was standard, I used the following information about body angle, distance, and position:

Angle 1: trunk tilt Angle, specifically the angle between the trunk (the line connecting the midpoint of the hip joint and the midpoint of the left and right shoulder joints) and the vertical axis;

Distance 1: knee-ankle distance, specifically the distance between the knee joint of the posterior leg and the ankle joint of the anterior leg.

Position 1: Posterior knee height, specifically the height of the knee joint of the posterior leg relative to the ankle joint of the anterior leg.

Scoring rules:

Trunk inclination Angle: There are two conditions for this angle: ① Standard angle: the trunk is

parallel to the vertical axis, and the range of this angle is [0, 5]. ② Non-standard angle: the trunk is not parallel to the vertical axis, the angle range is (5, +∞).

Knee-ankle distance: There are two kinds of distance: ① Standard distance: the distance between the knee joint of the back leg and the ankle joint of the front leg is [0, 2], in centimeters. ② Non-standard distance: the distance between the knee joint of the posterior leg and the ankle joint of the anterior leg was (2, +∞), in centimeters.

Posterior knee height: There are two conditions for this height: ① Standard height: in the vertical axis direction, the knee joint of the posterior leg and the ankle joint of the anterior leg are the same. In this case, the height difference between the two is [0, 2], unit centimeter. ② Non-standard height: in the vertical axis direction, the knee joint of the posterior leg is higher than the ankle joint of the anterior leg. In this case, the height difference between the two is (2, +∞), unit centimeter.

Rating:

The movement is scored on a four-point scale of 3, 2, 1, and 0 as follows:

Score 3 points: all angle information, distance information and position information must fully meet the standard;

Score 2: does not meet any one or more of the above human body angle and distance information when the posterior knee height meets the standard;

Score 1 point: as long as position 1 does not meet the criteria, score 1 point;

Score 0: the user feels pain.

To sum up, if you are a FMS tester, you first deep learn the above background knowledge and scoring rules, and then carefully reason about the angle information and position information of the following user actions:

Trunk parallel to vertical axis;

The knee-ankle distance was in line with the standard distance.

The posterior knee height met the standard.

The user had no pain.

Finally, you are asked to rate user actions. The output format is as follows:

1. **Movement score: 2 points.**

2. **Main body parts: trunk, left lower limb, right lower limb.**
3. **Evaluation criteria: the height of the posterior knee meets the standard; Trunk not parallel to vertical axis; The knee-ankle distance was in line with the standard range. The user had no pain.**
4. **Reason the user's angle and position information one by one to determine whether it meets the standard;**
   a) **trunk inclination: does not meet the standard; Reasoning process: the trunk is not parallel to the vertical axis;**
   b) **knee-ankle spacing: meet the standard; Reasoning process: the distance between the posterior leg knee joint and the anterior leg ankle joint conforms to the standard range;**
   c) **Posterior knee height: meet the standard; Reasoning process: The knee joint of the posterior leg is highly consistent with the ankle joint of the anterior leg and meets the criteria.**
5. **The user's body may have functional and joint flexibility problems.**

M06-Right Inline Lunge-Side view

Background knowledge:

We divided the human body into six parts: head, torso, left upper limb, right upper limb, left lower limb, and right lower limb. In order to better focus on the fine-grained performance of user actions. The Inline Lunge with the right leg in front is the third Movement of the Functional Movement Screen (FMS), with the right leg stepping forward. To determine whether the user's lunge was standard, I used the following information about body angle, distance, and position:

Angle 1: trunk tilt angle, specifically the angle between the trunk (the line connecting the midpoint of the hip joint and the midpoint of the left and right shoulder joints) and the vertical axis;

Distance 1: knee-ankle distance, specifically the distance between the knee joint of the posterior leg and the ankle joint of the anterior leg.

Position 1: Posterior knee height, specifically the height of the knee joint of the posterior leg relative to the ankle joint of the anterior leg.

Scoring rules:

Trunk inclination Angle: There are two conditions for this angle: ① Standard angle: the trunk is parallel to the vertical axis, and the range of this angle is [0, 5]. ② Non-standard angle: the trunk is not parallel to the vertical axis, the angle range is (5, +∞).

Knee-ankle distance: There are two kinds of distance: ① Standard distance: the distance between the knee joint of the back leg and the ankle joint of the front leg is [0, 2], in centimeters. ② Non-standard distance: the distance between the knee joint of the posterior leg and the ankle joint of the anterior leg was (2, +∞), in centimeters.

Posterior knee height: There are two conditions for this height: ① Standard height: in the vertical axis direction, the knee joint of the posterior leg and the ankle joint of the anterior leg are the same. In this case, the height difference between the two is [0, 2], unit centimeter. ② Non-standard height: in the vertical axis direction, the knee joint of the posterior leg is higher than the ankle joint of the anterior leg. In this case, the height difference between the two is (2, +∞), unit centimeter.

Rating:

The movement is scored on a four-point scale of 3, 2, 1, and 0 as follows:

Score 3 points: all angle information, distance information and position information must fully meet the standard;

Score 2: does not meet any one or more of the above human body angle and distance information when the posterior knee height meets the standard;

Score 1 point: as long as position 1 does not meet the criteria, score 1 point;

Score 0: the user feels pain.

To sum up, if you are a FMS tester, you first deep learn the above background knowledge and scoring rules, and then carefully reason about the angle information and position information of the following user actions:

Trunk parallel to vertical axis;

The knee-ankle distance was in line with the standard distance.

The posterior knee height met the standard.

The user had no pain.

Finally, you are asked to rate user actions. The output format is as follows:

1. Movement score: 2 points.
2. Main body parts: trunk, left lower limb, right lower limb.
3. Evaluation criteria: the height of the posterior knee meets the standard; Trunk not parallel to vertical axis; The knee-ankle distance was in line with the standard range. The user had no pain.
4. Reason the user's angle and position information one by one to determine whether it meets the standard;
    a) trunk inclination: does not meet the standard; Reasoning process: the trunk is not parallel to the vertical axis;
    b) knee-ankle spacing: meet the standard; Reasoning process: the distance between the posterior leg knee joint and the anterior leg ankle joint conforms to the standard range;
    c) Posterior knee height: meet the standard; Reasoning process: The knee joint of the posterior leg is highly consistent with the ankle joint of the anterior leg and meets the criteria.
5. The user's body may have functional and joint flexibility problems.

M07-Left hand in upper shoulder flexibility-Frontal view

Background knowledge:

We divided the human body into six parts: head, torso, left upper limb, right upper limb, left lower limb, and right lower limb. In order to better focus on the fine-grained performance of user actions. The left hand in the upper Shoulder Mobility is the fourth Movement of the Functional Movement Screen (FMS), and the left hand is raised up to the back of the head. To determine whether the user's shoulder flexibility is standard, I used the following body distance information:

Distance 1: wrist spacing, specifically the spacing between the left and right wrist joints.

Scoring rules:

Wrist spacing: there are three kinds of spacing: ① standard spacing: the left and right wrist spacing does not exceed the length of a palm; ② Non-standard spacing: the distance between the left and right wrists exceeds the length of one palm, but does not exceed 1.5 palm lengths; ③ Non-standard

spacing: the left and right wrists were more than 1.5 palm lengths.

Rating:

The movement is scored on a four-point scale of 3, 2, 1, and 0 as follows:

Score 3: the distance between the left and right wrists is no more than the length of a palm;

Score 2: the distance between the left and right wrists is more than a palm's length, but no more than 1.5 palm's length;

Score 1: the distance between the left and right wrists is more than 1.5 palm length;

Score 0: the user feels pain.

To sum up, if you are a FMS tester, you first deep learn the above background knowledge and scoring rules, and then carefully reason about the distance information of the following user actions: <u>The left and right wrists should not be separated by more than the length of a palm.</u>

Finally, you are asked to rate user actions. The output format is as follows:

1. **Movement score: 2 points.**
2. **Main body parts: left upper limb and right upper limb.**
3. **Scoring basis: the distance between the left and right wrists of the user belongs to the second case, that is, more than one palm length, but not more than 1.5 palm lengths.**
4. **Reasoning the user's location information one by one to determine whether it meets the standard:**
    a) **Wrist spacing: does not meet the standard; Inference procedure: The user's left and right wrists were separated by more than one palm length, but not more than 1.5 palm lengths.**
5. **Problems users may have.**

M08-Right hand in upper shoulder flexibility-Frontal view

Background knowledge:

We divided the human body into six parts: head, torso, left upper limb, right upper limb, left lower limb, and right lower limb. In order to better focus on the fine-grained performance of user actions.

The right hand in the upper Shoulder Mobility is the fourth Movement of the Functional Movement Screen (FMS), and the right hand is raised up to the back of the head. To determine whether the user's shoulder flexibility is standard, I used the following body distance information:

Distance 1: wrist spacing, specifically the spacing between the left and right wrist joints.

Scoring rules:

Wrist spacing: there are three kinds of spacing: ① standard spacing: the left and right wrist spacing does not exceed the length of a palm; ② Non-standard spacing: the distance between the left and right wrists exceeds the length of one palm, but does not exceed 1.5 palm lengths; ③ Non-standard spacing: the left and right wrists were more than 1.5 palm lengths.

Rating:

The movement is scored on a four-point scale of 3, 2, 1, and 0 as follows:

Score 3: the distance between the left and right wrists is no more than the length of a palm;

Score 2: the distance between the left and right wrists is more than a palm's length, but no more than 1.5 palm's length;

Score 1: the distance between the left and right wrists is more than 1.5 palm length;

Score 0: the user feels pain.

To sum up, if you are a FMS tester, you first deep learn the above background knowledge and scoring rules, and then carefully reason about the distance information of the following user actions:

The left and right wrists should not be separated by more than the length of a palm.

Finally, you are asked to rate user actions. The output format is as follows:

1. **Movement score: 2 points.**
2. **Main body parts: left upper limb and right upper limb.**
3. **Scoring basis: the distance between the left and right wrists of the user belongs to the second case, that is, more than one palm length, but not more than 1.5 palm lengths.**
4. **Reasoning the user's location information one by one to determine whether it meets the standard:**
    a) **Wrist spacing: does not meet the standard; Inference procedure: The user's left and**

right wrists were separated by more than one palm length, but not more than 1.5 palm lengths.

5. **Problems users may have.**

M09-Left Active Straight-Leg Raise-Front view

Background knowledge:

We divided the human body into six parts: head, torso, left upper limb, right upper limb, left lower limb, and right lower limb. In order to better focus on the fine-grained performance of user actions. The Active Straight Leg Raise of the left leg is the fifth maneuver of the Functional Movement Screen (FMS), in which the left leg is straight and raised upward. To determine whether the user's active straight leg raise was standard, I used the following body position information:

Position 1: Ankle drop point, specifically the position of the raised side ankle drop point on the ground leg.

Scoring rules:

Ankle drop point: there are three situations in this position: ① standard position: the drop point of the raised side of the ankle joint is in the middle of the thigh of the ground leg and above (near the head direction); ② Non-standard position: the drop point of the raised side ankle joint is between the middle point of the thigh of the ground leg and the knee joint point; ③ Non-standard position: the drop point of the raised side ankle joint is between the knee joint point of the ground leg and the ankle.

Rating:

The movement is scored on a four-point scale of 3, 2, 1, and 0 as follows:

Score 3: the lifting side of the ankle joint falls in the middle of the thigh of the ground leg and above, that is, close to the side of the head;

Score 2: the point of the raised side ankle joint is between the middle point of the thigh of the ground leg and the knee joint point;

Score 1: the lifting side of the ankle joint falls between the knee joint point of the ground leg and the ankle;

Score 0: the user feels pain.

To sum up, if you are a FMS tester, you first deep learn the above background knowledge and scoring rules, and then carefully reason about the location information of the following user actions: The raised side ankle drops between the mid-thigh point of the ground leg and the knee joint point.

Finally, you are asked to rate user actions. The output format is as follows:

1. **Movement score 2 points.**
2. **Main body parts: left lower limb and right lower limb.**
3. **Scoring basis: the user's ankle falls between the middle point of the thigh of the ground leg and the knee joint point.**
4. **Reasoning the user's location information one by one to determine whether it meets the standard:**
    a) **Position 1: does not meet the standard; Inference process: The user's ankle falls between the mid-thigh point of the ground leg and the knee joint point.**
5. **Problems users may have.**

M10-Right Active Straight-Leg Raise-Front view

Background knowledge:

We divided the human body into six parts: head, torso, left upper limb, right upper limb, left lower limb, and right lower limb. In order to better focus on the fine-grained performance of user actions. The Active Straight Leg Raise of the right leg is the fifth Movement of the Functional Movement Screen (FMS), in which the right leg is straight and raised upward. To determine whether the user's active straight leg raise was standard, I used the following body position information:

Position 1: Ankle drop point, specifically the position of the raised ankle joint drop point on the ground leg.

Scoring rules:

Ankle drop point: there are three situations in this position: ① standard position: the drop point of the raised side of the ankle joint is in the middle of the thigh of the ground leg and above (near the head direction); ② Non-standard position: the drop point of the raised side ankle joint is between the middle point of the thigh of the ground leg and the knee joint point; ③ Non-standard position:

the drop point of the raised side ankle joint is between the knee joint point of the ground leg and the ankle.

Rating:

The movement is scored on a four-point scale of 3, 2, 1, and 0 as follows:

Score 3: the lifting side of the ankle joint falls in the middle of the thigh of the ground leg and above, that is, close to the side of the head;

Score 2: the point of the raised side ankle joint is between the middle point of the thigh of the ground leg and the knee joint point;

Score 1: the lifting side of the ankle joint falls between the knee joint point of the ground leg and the ankle;

Score 0: the user feels pain.

To sum up, if you are a FMS tester, you first deep learn the above background knowledge and scoring rules, and then carefully reason about the location information of the following user actions: The raised side ankle drops between the mid-thigh point of the ground leg and the knee joint point.

Finally, you are asked to rate user actions. The output format is as follows:

1. **Movement score 2 points.**
2. **Main body parts: left lower limb and right lower limb.**
3. **Scoring basis: the user's ankle falls between the middle point of the thigh of the ground leg and the knee joint point.**
4. **Reasoning the user's location information one by one to determine whether it meets the standard:**
   a) **Position 1: does not meet the standard; Inference process: The user's ankle falls between the mid-thigh point of the ground leg and the knee joint point.**
5. **Problems users may have.**

M11-Trunk Stability Push-Up-Front view

Background knowledge:

We divided the human body into six parts: head, torso, left upper limb, right upper limb, left lower limb, and right lower limb. In order to better focus on the fine-grained performance of user actions. The Trunk Stability Push-Up is the sixth maneuver of the Functional Movement Screen (FMS). To determine whether a user was performing a standard trunk stability push-up, I used the following body angle and position information:

Position 1: Wrist position, specifically, the position relationship between wrist joint, nose tip and shoulder joint in the direction of the horizontal axis.

Angle 1: the trunk-lower limb angle, specifically the angle between the trunk (the line between the midpoint of the hip joint and the midpoint of the left and right shoulder joints) and the lower limb (the line between the hip joint and the ankle joint);

Angle 2: Lower limb inclination angle, specifically the thigh (hip joint and ankle joint connection line) and the horizontal plane angle.

Scoring rules:

Wrist position: there are three situations in this position: ① Standard position: in the direction of the horizontal axis, the wrist coincides with the tip of the nose or is on the right side of the tip of the nose, that is, close to the top of the head. ② Non-standard position: in the horizontal axis direction, the wrist is between the tip of the nose and the shoulder joint. ③ Non-standard position: in the horizontal axis direction, the wrist is on the left side of the shoulder joint point, that is, near the foot direction.

The Angle between trunk and lower limbs: there are two kinds of angle: ① Standard angle: the trunk is parallel to the lower limbs. In this case, the angle range is [0, 5], unit degree. ② Non-standard angle: the trunk is not parallel to the lower limbs, in this case, the angle range is (5, +∞), unit degree.

Lower limb inclination Angle: there are two kinds of angle: ① Standard angle: the angle between the thigh and the horizontal axis is (10, +∞), unit degree. ② Non-standard angle: the angle between the thigh and the horizontal axis ranges from [0 to 10], unit degree.

Rating:

The movement is scored on a four-point scale of 3, 2, 1, and 0 as follows:

Score 3: For male users, the wrist position is the standard position, the trunk-lower limb angle is the

standard angle, and the lower limb angle is the standard angle to get a full score of 3; For female users, the wrist position is the non-standard position, the trunk-lower limb angle is the standard angle, and the lower limb inclination angle is the standard angle to get a full score of 3;

Score 2: for male users, the wrist position is the non-standard position; ② the trunk-lower limb angle is the standard angle; For female users, the wrist position is the non-standard position ③, the trunk-lower limb angle is the standard angle, and the lower limb inclination angle is the standard angle.

Score 1: for male users, wrist position is non-standard position 2, lower limb angle is non-standard angle; For female users, the wrist position is the non-standard position ③, and the lower limb inclination angle is the non-standard angle.

Score 0: the user feels pain.

To sum up, if you are a FMS tester, you first deep learn the above background knowledge and scoring rules, and then carefully reason about the angle information and position information of the following male user's actions:

The wrist position was the standard position.

The angle between trunk and lower limbs was the standard angle.

The lower limb inclination angle was the standard angle.

Finally, you are asked to rate user actions. The output format is as follows:

1. **Movement score: 2 points.**
2. **Main body parts: left upper limb, right upper limb, head, trunk, left lower limb, right lower limb.**
3. **Scoring basis: the user is male; The wrist position was non-standard position ②, the trunk-lower limb angle was the standard angle, and the lower limb inclination angle was the standard angle.**
4. **Reasoning the user's location information one by one to determine whether it meets the standard:**
    a) **Position 1: does not meet the standard; Inference process: The wrist position did not coincide with or be on the right side of the nose tip;**

b) **Angle 1: meet the standard; Inference process: the Angle between trunk and lower limbs was the standard Angle, that is, the trunk was parallel to the lower limbs.**

c) **Angle 2: meet the standard; Inference procedure: The lower limb inclination Angle is the labeling Angle.**

5. **Problems users may have.**

M12-Rotary Stability (Ipsilateral&Left) -Front view

Background knowledge:

We divided the human body into six parts: head, torso, left upper limb, right upper limb, left lower limb, and right lower limb. In order to better focus on the fine-grained performance of user actions. Ipsilateral left Rotary Stability is the seventh maneuver of the Functional Movement Screen (FMS), which requires the left knee and ankle joints to be close to each other. To determine whether the user's action was standard, I used the following human distance information:

Distance 1: knee-ankle distance, specifically the distance between the left elbow joint and the left knee joint;

Scoring rules:

Knee-ankle distance: There are two kinds of distance: ① Standard distance: the distance range between the left elbow joint and the left knee joint is [0, 2], unit centimeter. ② Non-standard distance: the distance between the left elbow joint and the left knee joint was (2, +∞), unit centimeter.

Rating:

The movement is scored on a three-point scale of 3, 2, or 0, as follows:

Score 3 points: the distance of the user's action 1 meets the standard distance range;

Score 2 points: the distance 1 of the user's action does not meet the standard distance range;

Score 0: the user feels pain.

To sum up, if you are a FMS tester, you first deep learn the above background knowledge and scoring rules, and then carefully reason about the distance information of the following user actions:

Distance 1 meets the standard distance range;

Finally, you are asked to rate user actions. The output format is as follows:

1. **Movement score: 2 points.**
2. **Main body parts: left upper limb and left lower limb.**
3. **Scoring criteria: Distance 1 is the non-standard range.**
4. **Reasoning the user's location information one by one to determine whether it meets the standard:**
    a) **Distance 1: does not meet the standard distance; Inference process: The distance between the left elbow and the left knee of the user is beyond the standard distance range.**
5. **Problems users may have.**

M13-Rotary Stability (Ipsilateral&Right) -Front view

Background knowledge:

We divided the human body into six parts: head, torso, left upper limb, right upper limb, left lower limb, and right lower limb. In order to better focus on the fine-grained performance of user actions. Rotary Stability is the seventh maneuver of Functional Movement Screen (FMS), which requires the right side of the body to be close to each other. To determine whether the user's action was standard, I used the following human distance information:

Distance 1: knee-ankle distance, specifically the distance between the right elbow joint and the right knee joint;

Scoring rules:

Knee-ankle distance: There are two kinds of distance: ① Standard distance: the distance range between the right elbow joint and the right knee joint is [0, 2], unit centimeter. ② Non-standard distance: the distance between the right elbow joint and the right knee joint was (2, +∞), unit centimeter.

Rating:

The movement is scored on a three-point scale of 3, 2, or 0, as follows:

Score 3 points: the distance of the user's action 1 meets the standard distance range;

Score 2 points: the distance 1 of the user's action does not meet the standard distance range;

Score 0: the user feels pain.

To sum up, if you are a FMS tester, you first deep learn the above background knowledge and scoring rules, and then carefully reason about the distance information of the following user actions:

Distance 1 meets the standard distance range;

Finally, you are asked to rate user actions. The output format is as follows:

1. **Movement score: 2 points.**
2. **Main body parts: right upper limb and right lower limb.**
3. **Scoring criteria: Distance 1 is the non-standard range.**
4. **Reasoning the user's location information one by one to determine whether it meets the standard:**
    a) **Distance 1: does not meet the standard distance; Inference process: The distance between the user's right elbow and right knee is beyond the standard distance range.**
5. **Problems users may have.**

M14-Rotary Stability (Contralateral&Left upper limb) -Front view

Background knowledge:

We divided the human body into six parts: head, torso, left upper limb, right upper limb, left lower limb, and right lower limb. In order to better focus on the fine-grained performance of user actions. Rotary Stability of the left elbow and right knee on the opposite side of the body is the seventh maneuver of the Functional Movement Screen (FMS), which requires the left elbow and right knee to be close to each other. To determine whether the user's action was standard, I used the following human distance information:

Distance 1: knee-ankle distance, specifically the distance between the left elbow joint and the right

knee joint;

Scoring rules:

Knee-ankle distance: There are two kinds of distance: ① Standard distance: the distance range between the left elbow joint and the right knee joint is [0, 2], unit centimeter. ② Non-standard distance: the distance between the left elbow joint and the left knee joint was (2, +∞), unit centimeter.

Rating:

The movement is scored on a scale of 2, 1, or 0, as follows:

Score 2 points: the distance 1 of the user's action meets the standard distance range;

Score 1: the distance 1 of the user's action does not meet the standard distance range;

Score 0: the user feels pain.

To sum up, if you are a FMS tester, you first deep learn the above background knowledge and scoring rules, and then carefully reason about the distance information of the following user actions: <u>Distance 1 meets the standard distance range;</u>

Finally, you are asked to rate user actions. The output format is as follows:

1. **Movement score: 1.**
2. **Main body parts: left upper limb and right lower limb.**
3. **Scoring criteria: Distance 1 is the non-standard range.**
4. **Reasoning the user's location information one by one to determine whether it meets the standard:**
    a) **Distance 1: does not meet the standard distance; Inference process: The distance between the user's left elbow and right knee is beyond the standard distance range.**
5. **Problems users may have.**

M15-Rotary Stability (Contralateral&Right upper limb) -Front view

Background knowledge:

We divided the human body into six parts: head, torso, left upper limb, right upper limb, left lower limb, and right lower limb. In order to better focus on the fine-grained performance of user actions. Rotary Stability of the contralateral right elbow and left knee is the seventh maneuver of the Functional Movement Screen (FMS), which requires the right elbow and the left knee to be close to each other. To determine whether the user's action was standard, I used the following human distance information:

Distance 1: knee-ankle distance, specifically the distance between the right elbow joint and the left knee joint;

Scoring rules:

Knee-ankle distance: There are two kinds of distance: ① Standard distance: the distance range between the right elbow joint and the left knee joint is [0, 2], unit centimeter. ② Non-standard distance: the distance range between the right elbow joint and the left knee joint was (2, +∞), unit centimeter.

Rating:

The movement is scored on a scale of 2, 1, or 0, as follows:

Score 2 points: the distance 1 of the user's action meets the standard distance range;

Score 1: the distance 1 of the user's action does not meet the standard distance range;

Score 0: the user feels pain.

To sum up, if you are a FMS tester, you first deep learn the above background knowledge and scoring rules, and then carefully reason about the distance information of the following user actions:

<u>Distance 1 meets the standard distance range;</u>

Finally, you are asked to rate user actions. The output format is as follows:

1. **Movement score: 1.**
2. **Main body parts: right upper limb and left lower limb.**
3. **Scoring criteria: Distance 1 is the non-standard range.**
4. **Reasoning the user's location information one by one to determine whether it meets the**

standard:

a) **Distance 1: does not meet the standard distance; Inference process: The distance between the user's right elbow and left knee is beyond the standard distance range.**

5. **Problems users may have.**
