# Supplementary material for: LLM-FMS: A fine-grained dataset for functional movement screen action quality assessment
Source: PLoS One. 2025 Mar 11;20(3):e0313707. doi: 10.1371/journal.pone.0313707 (PMC11896072; doi:10.1371/journal.pone.0313707)
Supplement: Attachment — Submitted filename: A rebuttal letter.pdf [file pone.0313707.s006.pdf]

A rebuttal letter to the comments made by the reviewers

Xing Qing-Jun

School of Sport Science, Beijing Sport University

xqj_fitness@163.com

Dear editor,

Thank you for your valuable comments and suggestions. We appreciate the significance of your comments in guiding our research and improving the quality of our paper. We have carefully studied your feedback and made the necessary revisions accordingly. Revision records were kept for your and the reviewer's review of the revised manuscript (Revised Manuscript with Track Changes.docx), and a revised unmarked version of the manuscript was submitted at your request (Manuscript.docx). The following is a response to your and the reviewer's comments:

Journal Requirements:

When submitting your revision, we need you to address these additional requirements.

1. Please ensure that your manuscript meets PLOS ONE's style requirements, including those for file naming. The PLOS ONE style templates can be found at https://journals.plos.org/plosone/s/file?id=wjVg/PLOSOne_formatting_sample_main_body.pdf and https://journals.plos.org/plosone/s/file?id=ba62/PLOSOne_formatting_sample_title_authors_affiliations.pdf

Response: Thank you for your feedback and the opportunity to address the requirements for manuscript submission to PLOS ONE. We have carefully reviewed the style requirements and have made the necessary adjustments to ensure that our manuscript meets the journal's standards. We have followed the PLOS ONE style templates available at the provided links.

2. Thank you for stating the following financial disclosure: [This study has been supported by the National Natural Science Foundation of China, Grant No. 72071018, the

Fundamental Research Funds for the Central Universities of China, No. 2023GCZX003 (Research on the Nonlinear Accurate Measurement System of Exercise Loads)]. Please state what role the funders took in the study. If the funders had no role, please state: "The funders had no role in study design, data collection and analysis, decision to publish, or preparation of the manuscript." If this statement is not correct you must amend it as needed. Please include this amended Role of Funder statement in your cover letter; we will change the online submission form on your behalf

Response: Thank you for your feedback. The funder (Shen Yan-Fei) had role in study design. We have included this revised funder role statement in the cover letter.

3. Thank you for stating the following in the Acknowledgments Section of your manuscript: [This study has been supported by the National Natural Science Foundation of China, Grant No. 72071018, the Fundamental Research Funds for the Central Universities of China, No. 2023GCZX003 (Research on the Nonlinear Accurate Measurement System of Exercise Loads).] We note that you have provided funding information that is not currently declared in your Funding Statement. However, funding information should not appear in the Acknowledgments section or other areas of your manuscript. We will only publish funding information present in the Funding Statement section of the online submission form. Please remove any funding-related text from the manuscript and let us know how you would like to update your Funding Statement. Currently, your Funding Statement reads as follows: This study has been supported by the National Natural Science Foundation of China, Grant No. 72071018, the Fundamental Research Funds for the Central Universities of China, No. 2023GCZX003 (Research on the Nonlinear Accurate Measurement System of Exercise Loads)] Please include your amended statements within your cover letter; we will change the online submission form on your behalf.

Response: Thank you for your feedback. We have deleted the funding information in the manuscript and updated it in the cover letter.

4. Thank you for uploading your study's underlying data set. Unfortunately, the repository

you have noted in your Data Availability statement does not qualify as an acceptable data repository according to PLOS's standards. At this time, please upload the minimal data set necessary to replicate your study's findings to a stable, public repository (such as figshare or Dryad) and provide us with the relevant URLs, DOIs, or accession numbers that may be used to access these data. For a list of recommended repositories and additional information on PLOS standards for data deposition, please see https://journals.plos.org/plosone/s/recommended-repositories.

Response: Thank you for your feedback. We have uploaded the dataset to Figshare, here is the link to access it: https://doi.org/10.6084/m9.figshare.c.7601630.v1.

5. Please include captions for your Supporting Information files at the end of your manuscript, and update any in-text citations to match accordingly. Please see our Supporting Information guidelines for more information: http://journals.plos.org/plosone/s/supporting-information.

Response: Thank you for your feedback. We have included captions for all Supporting Information files at the end of our manuscript, ensuring that each caption matches the corresponding file name and number. Additionally, we have updated all in-text citations to accurately reflect these captions, maintaining consistency throughout the document.

6. Please review your reference list to ensure that it is complete and correct. If you have cited papers that have been retracted, please include the rationale for doing so in the manuscript text, or remove these references and replace them with relevant current references. Any changes to the reference list should be mentioned in the rebuttal letter that accompanies your revised manuscript. If you need to cite a retracted article, indicate the article’s retracted status in the References list and also include a citation and full reference for the retraction notice.

Response: Thank you for your important reminder regarding the review of our reference list. We have thoroughly checked our references to ensure their completeness and accuracy. We have also verified that none of the cited papers have been retracted. However, in the unlikely event that we find any retracted references in the future, we will

follow the journal's guidelines by either providing a rationale for their inclusion in the manuscript text or removing them and replacing them with relevant current references. If any changes are made to the reference list, we will certainly mention these changes in the rebuttal letter accompanying our revised manuscript.

Review Comments to the Author

Reviewer #1:

1. Abstract should higlight the probem statement and address the experimental results obtained.

Response: Thank you for your valuable feedback on our abstract. We have carefully revised the abstract to ensure that it clearly highlights the problem statement and addresses the experimental results obtained.

2. The structure of the manuscript needs to be mentioned.

Response: Thank you for your feedback regarding the structure of our manuscript. We have carefully reviewed the structure and have made the necessary adjustments to ensure that it aligns with the requirements of PLOS ONE, as outlined in the provided style templates:

PLOSOne_formatting_sample_main_body.pdf

PLOSOne_formatting_sample_title_authors_affiliations.pdf

We have also ensured that the manuscript is formatted according to PLOS ONE's style requirements, including font sizes, line spacing, and margins. Additionally, we have checked the file naming conventions and have made sure that all supporting information files are named appropriately and are referenced correctly within the manuscript.

3. Literature review should discuss the several work related to this field.

Response: Thank you for your suggestions on strengthening the literature review section of our manuscript. We have significantly revised the *Introduction*, focusing in particular on **the fourth and sixth paragraphs of the *Introduction***, to provide a more comprehensive discussion of related work in the field of intelligent evaluation of FMS and the application of LLMs in motion analysis, prediction, and control.

In the fourth paragraph of the introduction, we extend the discussion of intelligent evaluation of functional motor screens (FMS) using deep learning methods. We also added a new study from 2024 that explores the use of deep learning techniques for FMS

evaluation **[line number: 87-92]**. The following new articles were added:

[1] Lin X, Liu Y, Feng C, et al. Automatic Evaluation Method for Functional Movement Screening Based on Multi-Scale Lightweight 3D Convolution and an Encoder–Decoder[J]. Electronics, 2024, 13(10): 1813.

Furthermore, in paragraph VI, we present recent advances in the use of large language models (LLMs) for motion analysis, prediction, and control. In this paper, we discuss in detail a recent study of LLMs for action prediction in fine-grained videos, which demonstrated the effectiveness of LLMs for understanding human motion and videos, highlighting significant performance improvements over prior approaches **[line number: 101-115]**. This section now presents more clearly the state of the art of research in this area and the potential of LLMS. The following new articles were added:

[1] Zhao Q, Wang S, Zhang C, et al. Antgpt: Can large language models help long-term action anticipation from videos?[J]. arXiv preprint arXiv:2307.16368, 2023.

[2] Joublin F, Ceravola A, Smirnov P, et al. CoPAL: corrective planning of robot actions with large language models[C]//2024 IEEE International Conference on Robotics and Automation (ICRA). IEEE, 2024: 8664-8670.

4. Experiments should be done by comparing with several works.

Response: Thank you for your suggestion to compare our experimental results with several related works. We have addressed this by comparing the FMS coarse-grained action scoring results obtained using our LLM-based assessment framework with the results from several recent FMS action assessment studies. The comparison results are presented in Table 4, where we have evaluated the performance using three key metrics: Accuracy, macro F1-score (maF1), and kappa coefficient.

5. Result analysis needs to be explained elaborately.

Response: Thank you for your valuable feedback regarding the need for a more elaborate explanation of our result analysis. We reinterpret the results of the study, explaining the evaluation effect of the method on each action of FMS, and the performance comparison between the method of this study and other studies on the evaluation method of FMS action

rating. **[line number: 358-377].**

6. Future work needs to be discussed.

Response: Thank you for your suggestion to include a discussion on future work in our manuscript. We have addressed this by adding a dedicated section in the Discussion part of our paper, starting at **line [460-464]**, where we outline our plans for future research directions. In the future, we will leverage the contextual learning ability and logical reasoning ability of LLMs to realize the fine-grained action quality assessment research of more diverse sports. On the other hand, the action evaluation research based on LLMs multi-modal fusion can be explored with the help of LLMs' ability to understand the action behavior in images and videos.

Reviewer #2:

The overall weakness of the paper are as follows:

1. Limited theoretical grounding for the dataset's novelty and importance in the broader action quality assessment landscape.

Response: Thank you for your suggestion. In the first four paragraphs of the Introduction **[line number: 37-100]**, we discuss the theoretical basis of the dataset in the field of motion quality assessment, highlighting the importance of this dataset in the field of sports rehabilitation. In addition, we discuss the novelty of this dataset in the **Discussion** section **[line number: 380-414]**.

2. The lack of comparison with existing datasets and methods weakens the paper's positioning.

Response: Thank you for your suggestion. We add a comparison and discussion with existing action quality assessment datasets in the Discussion section **[line number: 380-435]** and a comparison with existing automated FMS assessment methods in the discussion section **[line number: 436-452]**, thus highlighting the novelty of the LLM-FMS dataset and the LLMs-based action assessment framework in this study.

Few recommendations:

1. Provide a more detailed literature review with explicit comparisons to existing datasets and methodologies.

Response: Thank you for your suggestion. We provide a more detailed literature review of research on automated FMS evaluation in the fourth introductory paragraph **[line number: 68-92]**, and a literature review of recent research on in-video action prediction and motion control with the aid of LLMs in the sixth paragraph **[line number: 101-115]**; A comparison of existing datasets **[line number: 380-403]** and methods **[line number: 436-452]** is also presented in the discussion section.

2. Include demographic and diversity statistics for participants in the dataset description.

Response: Thank you for your suggestion. In the "Basic information of the dataset" section of the "LLM-FMS fine-grained dataset" section of the Methods section **[line number: 194]**, the demographic and diversity statistics of the subjects of the dataset are explained. Specific statistical information is attached to S1 Table-Demographic Information of the subjects.
